# Supplementary material for: Fossil brains provide evidence of underwater feeding in early seals
Source: Commun Biol. 2023 Aug 17;6:747. doi: 10.1038/s42003-023-05135-z (PMC10435510; doi:10.1038/s42003-023-05135-z)
Supplement: Supplementary file 2 — Supplementary Information [file 42003_2023_5135_MOESM2_ESM.pdf]

Supplementary Information

Fossil brains provide evidence of underwater feeding in early seals

George A. Lyras[1], Lars Werdelin[2], Bartholomeus G.M. van der Geer[3], Alexandra A.E. van der Geer[4,5*]

[1] Faculty of Geology and Geoenvironment, Department of Historical Geology-Palaeontology, National and Kapodistrian University of Athens, 15784 Zografos, Greece (ORCID 0000-0002-4337-6708)

[2] Department of Palaeobiology, Swedish Museum of Natural History, SE-10405 Stockholm, Sweden (ORCID 0000-0002-9586-4017)

[3] Casa del Vento, 50060 FI Santa Brigida, Italy

[4] Vertebrate Evolution, Development and Ecology, Naturalis Biodiversity Center, 2333 RA Leiden, the Netherlands (ORCID 0000-0002-9588-4739)

[5] Institute of Biology, Leiden University, 2311 EZ Leiden, The Netherlands

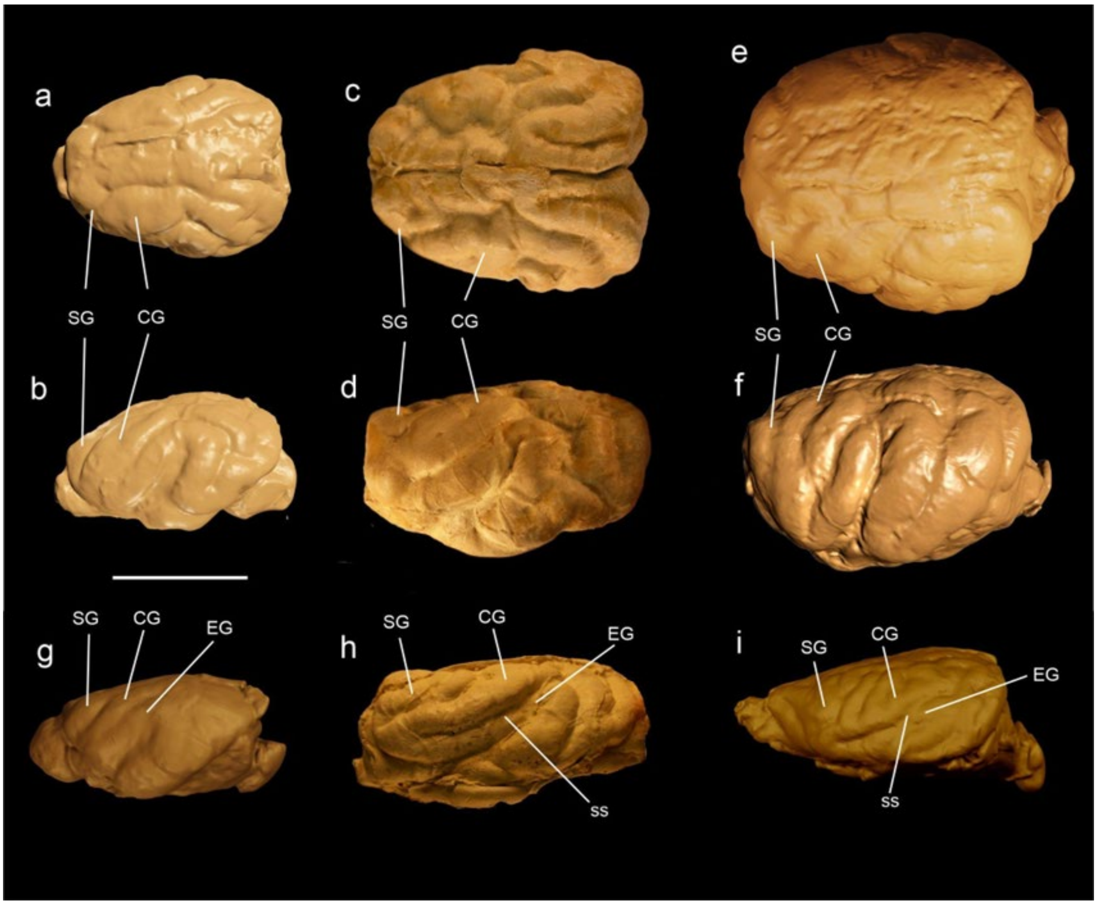

Supplementary Figure 1. Endocranial casts of stem pinnipeds and mustelids. a,b: Potamotherium valletoni (FMNH PM 58906); c,d: Enaliarctos sp. (FMNH PM 58944); e,f: Pinnarctidion bishopi (FMNH PM 57161); g: Promartes sp. (FMNH P 25233); h: Mionictis sp. (FMNH PM 58904); i: Lutra lutra (FMNH 75863). a,c and e: dorsal views. b,d, g, h and i: lateral views. SG: sigmoid gyrus, CG: coronal gyrus. EG: ectosylvian gyrus, ss: suprasylvian sulcus. Scale bar 3 cm

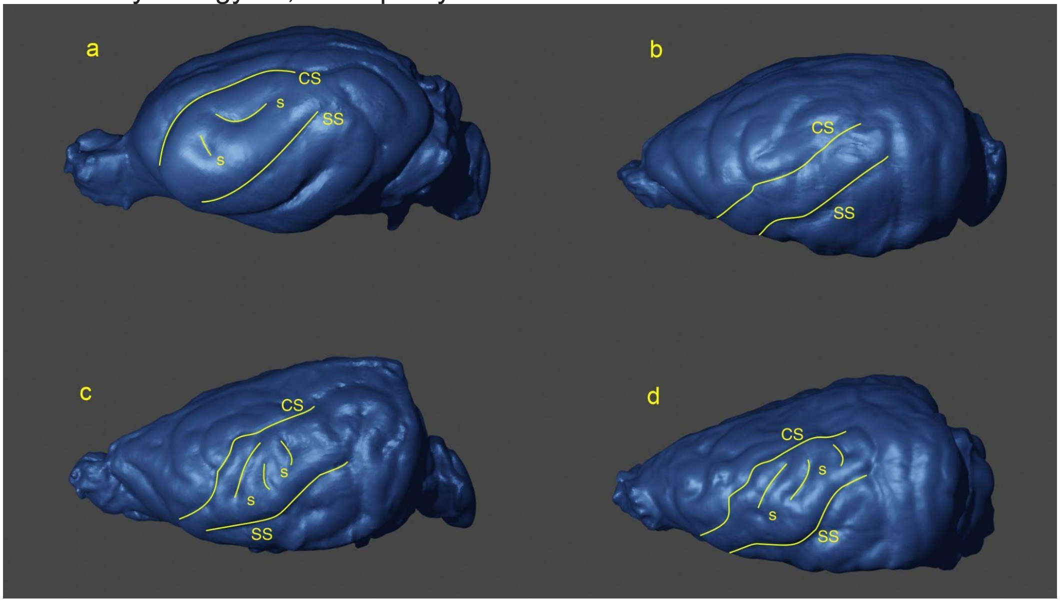

Supplementary Figure 2. Endocasts of four extant carnivores discussed in the text. (a) *Cynogale bennettii,* (b) *Aonyx capensis*, (c) *Lutra lutra,* (d) *Lontra canadensis.* Abbreviations: CS, coronal sulcus; SS, suprasylvian sulcus; s, secondary sulcus.

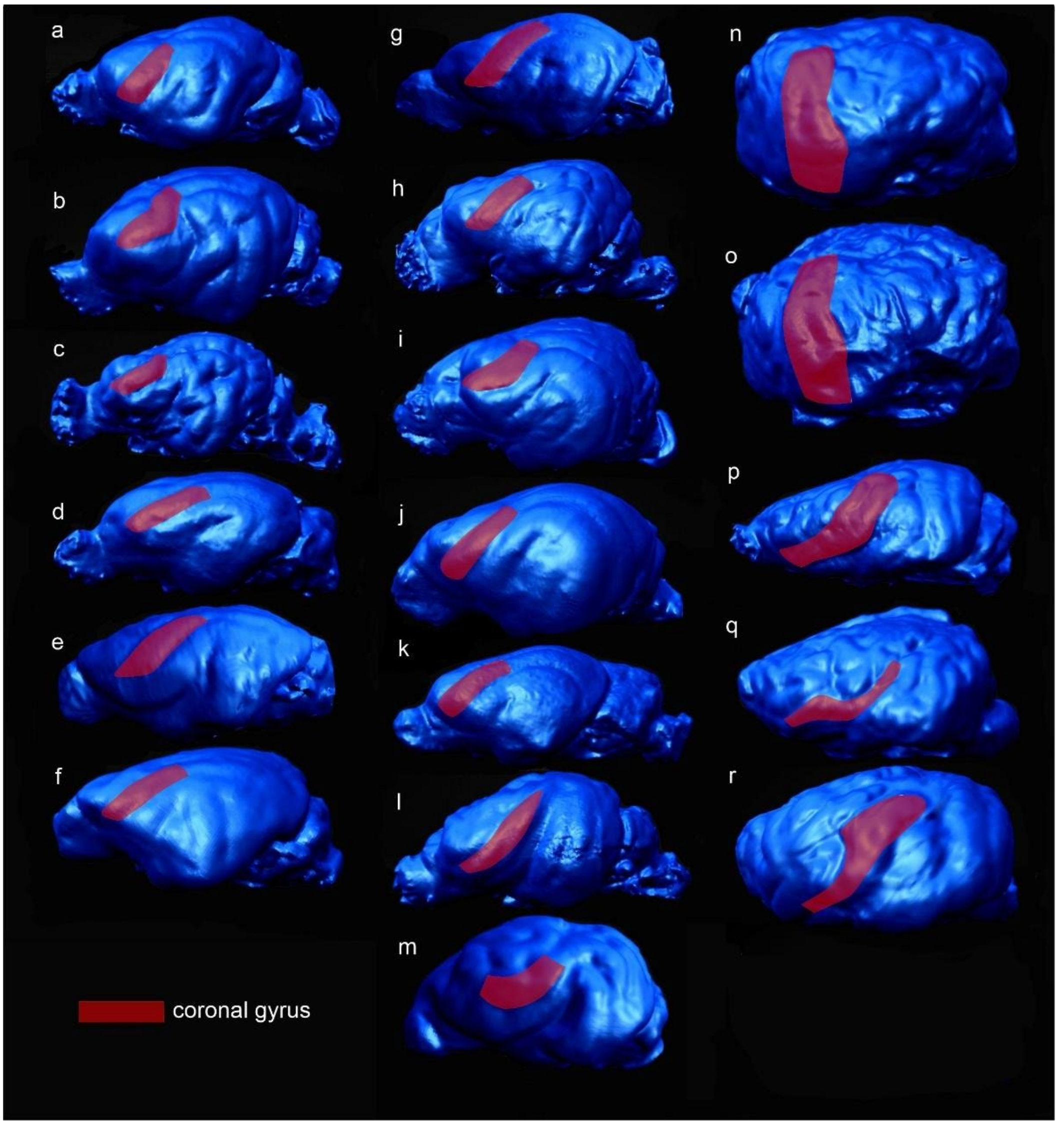

Supplementary Figure 3. Endocasts of carnivoran taxa mentioned, but not depicted in Fig. 2 of the main text. The endocasts are seen from lateral view. The shaded area indicates the coronal gyrus. a: *Proteles cristata*; b: *Neofelis nebulosa*; c: *Panthera tigris*; d: *Arctogalidia trivirgata*; e: *Atilax paludinosus*; f: *Cynictis penicillata*; g: *Fossa fossana*; h: *Cuon alpinus*; i: *Canis mesomelas*; j: *Vulpes zerda*; k: *Prionodon pardicolor*; l: *Ursus thibetanus*; m: *Ailurus fulgens*; n: *Pusa hispida*; o: *Halichoerus grypus*; p: *Lontra canadensis*; q: *Enhydra lutris*; r: *Aonyx capensis*. All specimens are drawn to the same anteroposterior length. See Supplementary Data for specimen accession numbers.

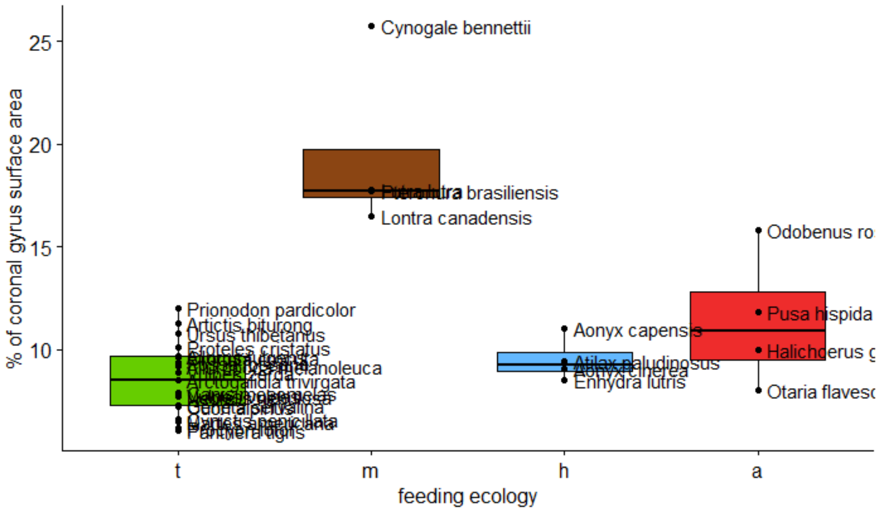

Supplementary Figure 4. Boxplots of the relative size of the coronal gyrus among carnivorans with different feeding ecologies. t: terrestrial feeding; h: hand- orientated foraging/capturing, semi-aquatic feeding (where prey is captured underwater but processed in air); m: mouth-orientated foraging/capturing, semi-aquatic feeding; a: aquatic or mostly aquatic feeding (pinnipeds). The midline represents the median.

.

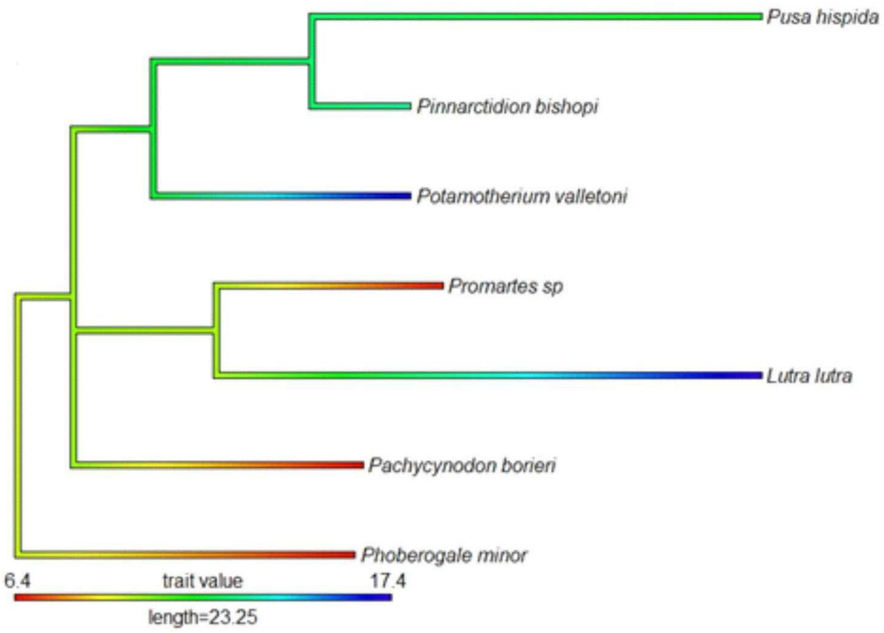

Supplementary Figure 5. Ancestral state reconstruction of the percentage of superficially exposed surface area of the coronal gyrus compared to the total area of superficially exposed cerebral cortex (cladogram in Fig. 3).

Supplementary Table 1. Permutation test and pairwise comparisons.

**Permutation test**	**χ^2 = 21.945, df = 3, p = 0.00067**		
**Pairwise comparisons**	**Stat**	**p.value**	**p.adjust**
mouth orientated – terrestrial	-4.145	0.00003	**0.0002035**
hand orientated- terrestrial	-1.028	0.3041	0.3041000
terrestrial – aquatic	-2.255	0.02415	**0.0483000**
mouth orientated – hand orientated	2.328	0.01993	**0.0483000**
mouth orientated – aquatic	2.044	0.04094	0.0614100
hand orientated – aquatic	-1.07	0.2847	0.3041000

Abbreviation: df, degrees of freedom; Stat, statistics. Significant p-values are highlighted in bold.

Supplementary Table 2. Data of fossil taxa used in this study. AMNH: American Museum of Natural History (New York); FMNH: Field Museum of Natural History (Chicago); LACM: Natural History Museum of Los Angeles County (Los Angeles); NMB: Naturhistorisches Museum (Basel), MNHN: Muséum National d'Histoire Naturelle (Paris), NHMW: Naturhistorisches Museum Wien (Vienna); UOMNH: Museum of Natural and Cultural History, University of Oregon (Eugene).

**Taxon**	**Specimen accession number**	**Stratigraphic age**	**Locality**
Enaliarctos sp.	FMNH PM 58944 (plaster copy of UOMNH 26269)	Late Oligocene-Early Miocene	Oregon, USA
Mionictis sp.	FMNH PM 58904 (latex endocast from F:AM 63296)	Middle- Late Miocene (Clarendonian)	MacAdams Quarry Donley, Texas, USA
Pachycynodon boriei	MNHN nn (plaster endocast)	Oligocene	Phosphorites, Quercy, France
Phoberogale minor	FMNH PM 59026 (plaster endocast from NHMW A 4445)	Oligocene	Bach, Quercy, France
Pinnarctidion bishopi	FMNH PM 57161 (plaster copy of LACM 5302)	Early Miocene (Arikareean)	Pyramid Hill, Kern, California, USA
Potamotherium valletoni	FMNH PM 58906 (endocast made from AMNH 22520*)	Early Miocene (Aquitanian)	Allier, France
Potamotherium valletoni	NHM Sau 2280	Early Miocene (Aquitanian)	Saulcet, Allier, France
Promartes sp.	FMNH P 25233 (plaster endocast)	Early Miocene	Wounded Knee, U. Rosebud, South Dakota, USA

* There are several *Potamotherium* specimens in AMNH bearing the number 22520, including a partially exposed natural endocast, all coming from the same locality. In this study we used an endocast prepared from a sectioned partial skull.
