# Supplementary material for: Fossil brains provide evidence of underwater feeding in early seals
Source: Commun Biol. 2023 Aug 17;6:747. doi: 10.1038/s42003-023-05135-z (PMC10435510; doi:10.1038/s42003-023-05135-z)
Supplement: Supplementary file 3 — Description of Additional Supplementary Files [file 42003_2023_5135_MOESM3_ESM.pdf]

Description of Additional Supplementary Files

File name: Supplementary Data 1

Description: The source data on coronal gyrus area behind bar plots in the paper.
