# Supplementary material for: Fossil brains provide evidence of underwater feeding in early seals
Source: Commun Biol. 2023 Aug 17;6:747. doi: 10.1038/s42003-023-05135-z (PMC10435510; doi:10.1038/s42003-023-05135-z)
Supplement: Supplementary file 5 — Reporting Summary [file 42003_2023_5135_MOESM5_ESM.pdf]

nature portfolio

Corresponding author(s): Alexandra van der Geer

Last updated by author(s): Feb 9, 2023

Reporting Summary

Nature Portfolio wishes to improve the reproducibility of the work that we publish. This form provides structure for consistency and transparency in reporting. For further information on Nature Portfolio policies, see our Editorial Policies and the Editorial Policy Checklist.

Statistics

For all statistical analyses, confirm that the following items are present in the figure legend, table legend, main text, or Methods section.

n/a	Confirmed	
☒	☐	The exact sample size (*n*) for each experimental group/condition, given as a discrete number and unit of measurement
☒	☐	A statement on whether measurements were taken from distinct samples or whether the same sample was measured repeatedly
☒	☐	The statistical test(s) used AND whether they are one- or two-sided<br>*Only common tests should be described solely by name; describe more complex techniques in the Methods section.*
☒	☐	A description of all covariates tested
☒	☐	A description of any assumptions or corrections, such as tests of normality and adjustment for multiple comparisons
☒	☐	A full description of the statistical parameters including central tendency (e.g. means) or other basic estimates (e.g. regression coefficient) AND variation (e.g. standard deviation) or associated estimates of uncertainty (e.g. confidence intervals)
☒	☐	For null hypothesis testing, the test statistic (e.g. *F*, *t*, *r*) with confidence intervals, effect sizes, degrees of freedom and *P* value noted<br>*Give P values as exact values whenever suitable.*
☒	☐	For Bayesian analysis, information on the choice of priors and Markov chain Monte Carlo settings
☒	☐	For hierarchical and complex designs, identification of the appropriate level for tests and full reporting of outcomes
☒	☐	Estimates of effect sizes (e.g. Cohen's *d*, Pearson's *r*), indicating how they were calculated

Our web collection on statistics for biologists contains articles on many of the points above.

Software and code

Policy information about availability of computer code

Data collection	Surface scans (Next Engine) were converted into closed mesh models and imported into Blender version 3.1.0 for surface area measurements
Data analysis	No software or code was used to analyse the data

For manuscripts utilizing custom algorithms or software that are central to the research but not yet described in published literature, software must be made available to editors and reviewers. We strongly encourage code deposition in a community repository (e.g. GitHub). See the Nature Portfolio guidelines for submitting code & software for further information.

Data

Policy information about availability of data

All manuscripts must include a data availability statement. This statement should provide the following information, where applicable:

- Accession codes, unique identifiers, or web links for publicly available datasets
- A description of any restrictions on data availability
- For clinical datasets or third party data, please ensure that the statement adheres to our policy

All data are available in the Figures and Supporting Tables.

Human research participants

Policy information about studies involving human research participants and Sex and Gender in Research.

Reporting on sex and gender	N/A
Population characteristics	N/A
Recruitment	N/A
Ethics oversight	N/A

Note that full information on the approval of the study protocol must also be provided in the manuscript.

Field-specific reporting

Please select the one below that is the best fit for your research. If you are not sure, read the appropriate sections before making your selection.

☐ Life sciences ☐ Behavioural & social sciences ☒ Ecological, evolutionary & environmental sciences

For a reference copy of the document with all sections, see nature.com/documents/nr-reporting-summary-flat.pdf

Ecological, evolutionary & environmental sciences study design

All studies must disclose on these points even when the disclosure is negative.

Study description	We reconstruct the underwater foraging behaviour of one of the earliest pinnipeds (Potamotherium), in particular how it used its whiskers (vibrissae). For this, we analysed the coronal gyrus of the brain. This region receives somatosensory input from the head, including the whiskers.
Research sample	Our sample includes 7 fossil and 32 extant carnivorans
Sampling strategy	The fossil sample is limited to Carnivora species of which a complete endocast is preservedsample represents the phylogenetic tree of Carnivora including early forms of various clades of which a complete endocast is preserved.
Data collection	Surface area of the coronal gyras was calculated based on scanned endocasts
Timing and spatial scale	Scans were acquired in 2015 (July-August; Chicago), 2017 (Jan 29-Feb 5; Paris) and 2022 (May 23-28; Paris). Scans were processed and data collected in 2022 (June, July; Athens).
Data exclusions	No data was excluded
Reproducibility	Not applicable
Randomization	The fossil samples were not randomly chosen but based on the availability of preserved, complete endocasts. The extant samples were not randomly chosen but represent the major groups within Carnivora; groups were equally sampled as far as possible.
Blinding	Not applicable

Did the study involve field work? ☐ Yes ☒ No

Reporting for specific materials, systems and methods

We require information from authors about some types of materials, experimental systems and methods used in many studies. Here, indicate whether each material, system or method listed is relevant to your study. If you are not sure if a list item applies to your research, read the appropriate section before selecting a response.

Materials & experimental systems

n/a	Involved in the study
☒	☐ Antibodies
☒	☐ Eukaryotic cell lines
☐	☒ Palaeontology and archaeology
☒	☐ Animals and other organisms
☒	☐ Clinical data
☒	☐ Dual use research of concern

Methods

n/a	Involved in the study
☒	☐ ChIP-seq
☒	☐ Flow cytometry
☒	☐ MRI-based neuroimaging

Palaeontology and Archaeology

Specimen provenance

We used specimens from various natural history museums (see next item); the fossils are from the US and France. Permission to use the specimens in their care was given by the respective curators. These are Lawrence Heaney, William Simpson, and the late Bill Stanley (2015; Field Museum of Natural History, Chicago, USA), John Flynn, Jin Meng and Judy Galkin (2015; American Museum of Natural History, New York, USA), Christine Argot, Christine Lefèvre and Géraldine Veron (2017, 2022; Muséum National d'Histoire Naturelle, Paris, France)

Specimen deposition

Field Museum of Natural History (Chicago, USA), American Museum of Natural History (New York, USA), Muséum National d'Histoire Naturelle (Paris, France), National and Kapodistrian University of Athens, Department of Zoology (Greece)

Dating methods

Not applicable

☒ Tick this box to confirm that the raw and calibrated dates are available in the paper or in Supplementary Information.

Ethics oversight

No ethical aprovement was needed because the specimens are fossils, curated in public institutions.

Note that full information on the approval of the study protocol must also be provided in the manuscript.
